# Efficacy of CP-COV03 (a niclosamide-based inorganic nanohybrid product) against severe fever with thrombocytopenia syndrome virus in an *in vitro* model

**DOI:** 10.1128/spectrum.01399-24

**Published:** 2024-10-15

**Authors:** Min Han, Youn-Jung Lee, Sang Min Ahn, Jae Eun Seong, Jung Ah Lee, Yong Seop Lee, Jung Ho Kim, Jin Young Ahn, Su Jin Jeong, Nam Su Ku, Joon Sup Yeom, Jun Yong Choi

**Affiliations:** 1 Division of Infectious Diseases, Department of Internal Medicine, Yonsei University College of Medicine, Seoul, South Korea; 2 AIDS Research Institute, Yonsei University College of Medicine, Seoul, South Korea; National Taiwan University, Taipei, Taiwan

**Keywords:** SFTS, niclosamide, CP-COV03

## Abstract

Severe fever with thrombocytopenia syndrome (SFTS) is a tick-borne infectious disease caused by the SFTS virus (SFTSV). CP-COV03 is a novel antiviral candidate that significantly enhanced the bioavailability of niclosamide through inorganic-based drug delivery technology. The active pharmaceutical ingredient of CP-COV03, niclosamide, has been previously shown to possess broad-spectrum antiviral activity against over 30 different viruses in the *in vitro* tests. The aim of this study is to confirm the antiviral activity of CP-COV03 against the SFTSV in an *in vitro* model. Vero cells and SFTS viral stock NCCP43270, a 2015 Gangwon Province isolate, were used to obtain the 50% tissue culture infective dose of the virus. Vero cells seeded in 96-well plates were infected with SFTSV for 1 h. SFTSV-infected cells were treated with CP-COV03 at various concentrations of 0.1–100 μM and incubated for 7 days. On the seventh day of the culture, the cytopathic effect (CPE) of SFTSV was checked by microscopy and the cell viability was checked by using Cell Counting Kit-8 assay. The CPE reduced as the CP-COV03 concentration increased. The 50% inhibitory concentration (IC_50_) range of CP-COV03 was below 0.125 µM, as determined from the viral titers of culture supernatants collected on the third day posttreatment of CP-COV03. The plaque reduction assay showed that the IC_50_ of CP-COV03 was 1.893 µM, as determined from the percentage reduction of plaque counts for each drug concentration on the second day posttreatment with CP-COV03. This study suggests that CP-COV03 could be used as a potential antiviral agent for SFTS.

**IMPORTANCE:**

We demonstrated a concentration-dependent response and identified low a IC_50_ of CP-COV03. This result is comparable to other antiviral drugs used against viruses like severe acute respiratory syndrome coronavirus 2 (SARS-CoV-2). We believe that our study makes a significant contribution to the literature as our findings suggest that CP-COV03 may serve as a potential treatment for SFTS, highlighting its importance in the field of antiviral research.

## INTRODUCTION

Severe fever with thrombocytopenia syndrome (SFTS) is a tick-borne infectious disease caused by the SFTS virus (SFTSV), which belongs to the genus *Bandavirus* and family *Phenuiviridae* of the order *Bunyavirales*. It is characterized by fever, leukopenia, thrombocytopenia, and gastrointestinal symptoms. The outbreak was first reported in China in 2007 and subsequently in several other Asian countries, including South Korea, Japan, Vietnam, and Myanmar, with a fatality rate of 12–50% (1–3).

Given its endemic presence and high fatality rate in Asia, developing antiviral drugs for SFTSV is an urgent need. Several antiviral agents have been investigated for their efficacy against SFTS. Ribavirin is an RNA-dependent RNA polymerase inhibitor that emerged as a potential antiviral drug for SFTSV. However, results were conflicting; ribavirin showed concentration-dependent antiviral activity using an *in vitro* model but ribavirin treatment showed no significant effect on the platelet counts or viral loads, according to clinical data (4, 5).

Niclosamide has a long history of use as an FDA-approved anti-parasitic drug against *Taenia solium*, *Taenia saginata*, and *Diphyllobothrium latum*. It affects multiple signaling pathways and is currently being actively studied as a treatment for cancer and metabolic syndromes, with clinical studies underway (6).

Antiviral efficacy of niclosamide has been reported from 2004 to the present for 33 viruses that affect humans, including SARS-CoV, Middle East respiratory syndrome coronavirus (MERS-CoV), Zika, hepatitis C virus (HCV), and recently, SARS-CoV-2 (7–12). Particularly for SARS-CoV-2, it has shown better *in vitro* potency than remdesivir (13).

Remarkably, the mechanism underlying the broad-spectrum antiviral efficacy of niclosamide beyond the viral family was not elucidated until 2019 when Gassen et al., discovered that niclosamide activates autophagy via S-phase kinase-associated protein 2 (SKP2) inhibition (14, 15). Furthermore, this study provides mechanistic insights into the broad antiviral effects of niclosamide because cells eliminate viruses through autophagy. The study revealed that SARS-CoV-2 reduces autophagy through distinct viral proteins, leading to autophagosome–lysosome fusion blockade. However, niclosamide contributes to the activation of autophagy leading to viral degradation. Prevention of viral entry by altering endosomal pH and prevention of viral replication by inhibition of autophagy are plausible mechanisms of action (MOA) of niclosamide against viral infection (16).

Although niclosamide is expected to have broad-spectrum antiviral effects, its antiviral efficacy against SFTSV has not been experimentally confirmed. Thus, this study aimed to determine the antiviral efficacy of niclosamide against SFTSV.

Despite its broad antiviral efficacy, niclosamide has drawbacks, such as low solubility and bioavailability. However, recent clinical trials have demonstrated the safety of CP-COV03 using an organic-inorganic hybrid system, which overcomes these drawbacks (17). CP-COV03 is a niclosamide-based inorganic nanohybrid product engineered with MgO and hydroxypropyl methylcellulose (HPMC), forming hydrophilic niclosamide-MgO–HPMC (17). A previous study demonstrated that this product improved intestinal permeability without altering niclosamide metabolism (17). A phase 1 trial investigated the safety, tolerability, and pharmacokinetic characteristics of this product in healthy adult participants. The nanohybrid oral drug was well-tolerated and showed four times higher bioavailability compared to the commercially available niclosamide product, Yomesan. This drug delivery technology addresses the low bioavailability of niclosamide in the human body and enables the fine-tuning of plasma drug concentrations by adjusting the ratio of inorganic and organic excipients (18). This technology allows the development of antiviral agents tailored to virus inhibition concentration ( 50% inhibitory concentration [IC_50_]) values identified through cell experiments. Therefore, once the IC_50_ values of niclosamide, the active pharmaceutical ingredient (API) of CP-COV03, for various viruses are determined, antiviral agents can be rapidly developed based on this information. In other words, elucidating the antiviral efficacy of niclosamide, the API of CP-COV03, against SFTSV could accelerate the development of treatments for SFTS, a disease that currently lacks a cure.

## MATERIALS AND METHODS

### Cell lines, viruses, and compounds

Vero cells (ATCC no. CCL-81) were cultured in a Dulbecco’s modified Eagle’s medium (DMEM) (Lonza, Walkersville, MD, USA) containing 2% fetal bovine serum and 1% penicillin-streptomycin (Gibco, Carlsbad, CA, USA). The cells were incubated at 37°C in a humidified chamber containing 5% CO_2_. The NCCP43270 SFTS viral stock (an isolate from Gangwon Province in 2015) was used. The 50% tissue culture infective dose (TCID_50_) was calculated as described by Reed and Muench (19). CP-COV03 was dissolved in dimethyl sulfoxide (DMSO) to prepare a stock solution and diluted in a DMEM to prepare a working concentration before use.

### Cell viability assay

A Cell Counting Kit-8 (CCK-8; Sigma-Aldrich, USA) assay was performed to determine the cellular toxicity of CP-COV03. Vero cells (1 × 10^5^ cells/mL) were seeded into 96-well tissue culture plates and incubated overnight. Increasing concentrations of CP-COV03 (0–100 µM) in DMEM were added to each well. Thereafter, optical densities were measured at 450 nm using a VersaMax microplate reader (Molecular Devices, Sunnyvale, CA, USA).

### Antiviral assay

Vero cells were seeded into 96-well plates. In the biosafety level 3 experimental area, Vero cells were inoculated with 100 TCID_50_ of SFTSV for 1 h and the inoculum was removed. The cells were treated in quadruplicate with different CP-COV03 concentrations (0–100 µM) and incubated for 3 and 7 days.

The cytopathic effect (CPE) was observed under a 10 × 10 microscope on the seventh day of incubation. After staining the Vero cells with CCK-8 reagent, the absorbance (450 nm, reference: 650 nm) was measured to determine the antiviral activity of CP-COV03.

On the third day of incubation, a real-time quantitative polymerase chain reaction (RT-qPCR) was performed on the culture supernatant to quantify the SFTSV RNA load. RNA was extracted from the supernatant of samples treated with CP-COV03 using the QIAamp Viral RNA Kit (Qiagen, Hilden, Germany). The L-segment of the SFTSV was amplified using the GoTaq Probe one-step RT-qPCR system (Promega, Madison, WI, USA) to obtain the cycle threshold (Ct) value, which was then converted to a viral titer. The cutoff Ct-value for positive samples was set to 35 cycles. Serial 10-fold dilutions from 10^−7^ to 10^–2^ TCID_50_/mL of SFTSV transcript RNAs were used to generate a standard real-time reverse transcription polymerase chain reaction (RT-PCR) curve. The Ct values of viral RNA titers in the samples were obtained, and viral loads were calculated by transforming the Ct values in terms of the standard curve using Bio-Rad CFX Maestro software. Viral titers were expressed as log_10_ TCID_50_/mL (standard error of the mean).

Plaque reduction assay was performed as previously described, with modifications. Briefly, Vero cells were seeded in 96-well plates on the day before the assay was performed. After 24 h of incubation, Vero cells were inoculated with a twofold concentration of SFTSV at 100 TCID50 for 1 h. Cells were then treated with an equal amount added to the twofold concentration, resulting in a final CP-COV03 concentration ranging from 0 to 100 µM for 1 h at 37°C in 5% CO_2_. Unbound viral particles were removed by aspiration of the media, and the overlay medium was replaced with 100 µL of 2% fetal bovine serum (FBS) DMEM containing 1.5% carboxymethyl cellulose. The culture medium was used as a control. The cells were incubated for an additional 2 days. After 2 days of incubation, cells were fixed with 4% paraformaldehyde, incubated with 100 µL/well, and then blocked with 1% bovine serum albumin (BSA) and phosphate-buffered saline (PBS) with 0.1% Tween^®^ 20 Detergent (TBST) (Sigma-Aldrich). Cells were then incubated with mouse anti-SFTSV nucleoprotein antibody (Native Antigen Company) diluted with TBST in PBS for 60 min at room temperature, followed by incubation with horseradish peroxidase (HRP)-conjugated secondary antibodies. Visualization of SFTSV-infected cell foci was performed using a tetramethylbenzidine (TMB)-stabilized substrate for HRP (Promega), and the number of plaques was counted using the Immunospot reader. The percentage of plaque inhibition relative to the control wells (i.e., without the addition of CP-COV03) was determined for each drug concentration. IC_50_ was calculated using a Sigma Plot (SPSS Inc.) in an Excel add-in ED50V10.

## RESULTS

### Effects of CP-COV-03 on cell viability

**Fig 1:**
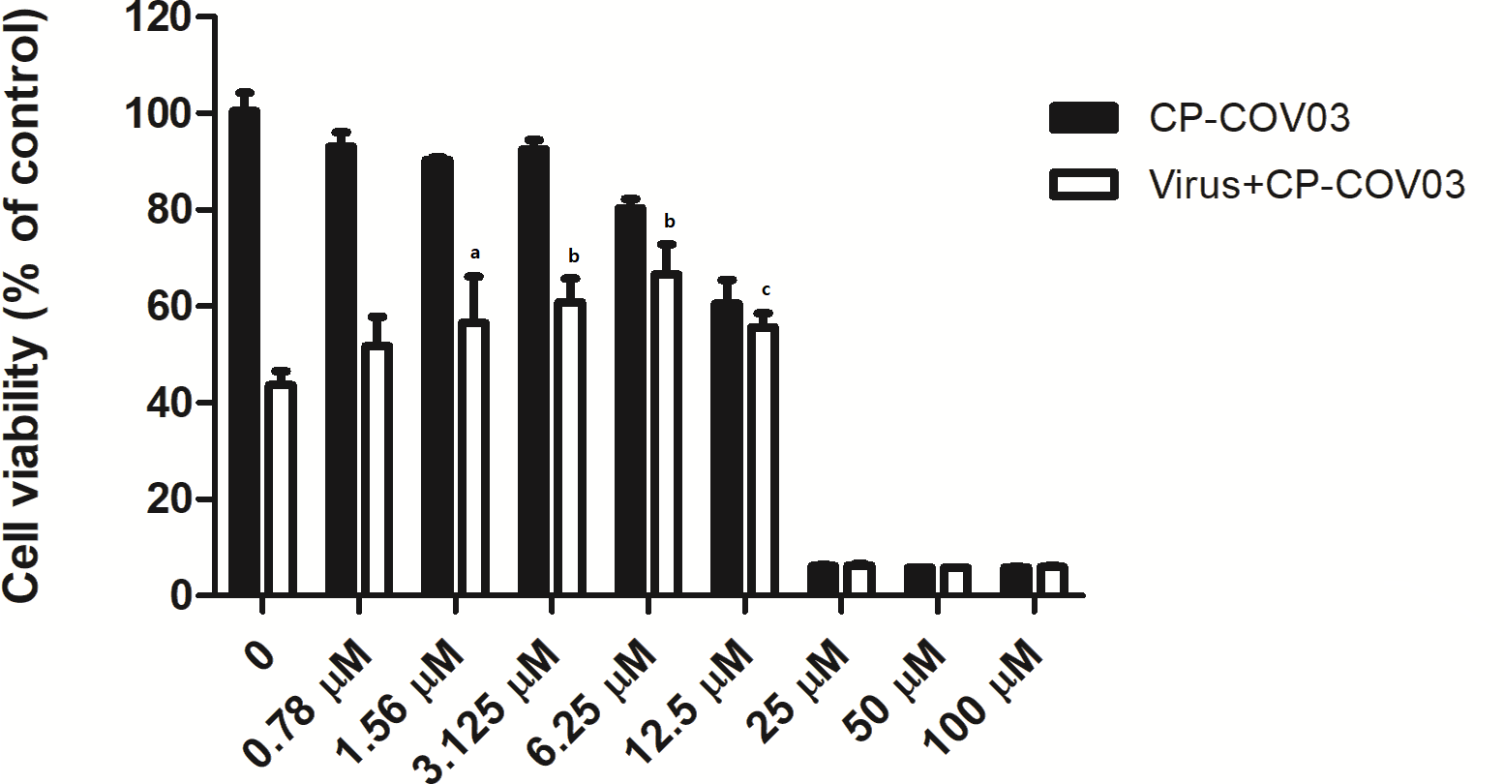
Effect of CP-COV03 on cell viability of Vero cells using CCK assay. CCK assay was done on the seventh day of posttreatment. The black bar represents the cell viability of uninfected Vero cells with CP-COV03. Cell viability decreased at CP-COV03 concentrations ≥25 µM resulting in cellular toxicity of CP-COV03. The white bar represents the cell viability of SFTSV-infected Vero cells with CP-COV03. As the concentration of CP-COV03 increased, cell viability also significantly increased. ^a^*p* < 0.05, ^b^*p* < 0.001, and ^c^*p* < 0.01 by one-way analysis of variance.

No significant decrease was observed in cell viability of CP-COV03-treated Vero cells at CP-COV03 concentrations ≤12.5 µM on the seventh day posttreatment (Fig. 1). In contrast, cell viability was decreased at CP-COV03 concentrations ≥25 µM on the seventh day posttreatment. Based on these results, we performed antiviral assays using noncytotoxic CP-COV03 concentrations ranging from 0 to 12.5 µM.

### Antiviral activity

**Fig 2:**
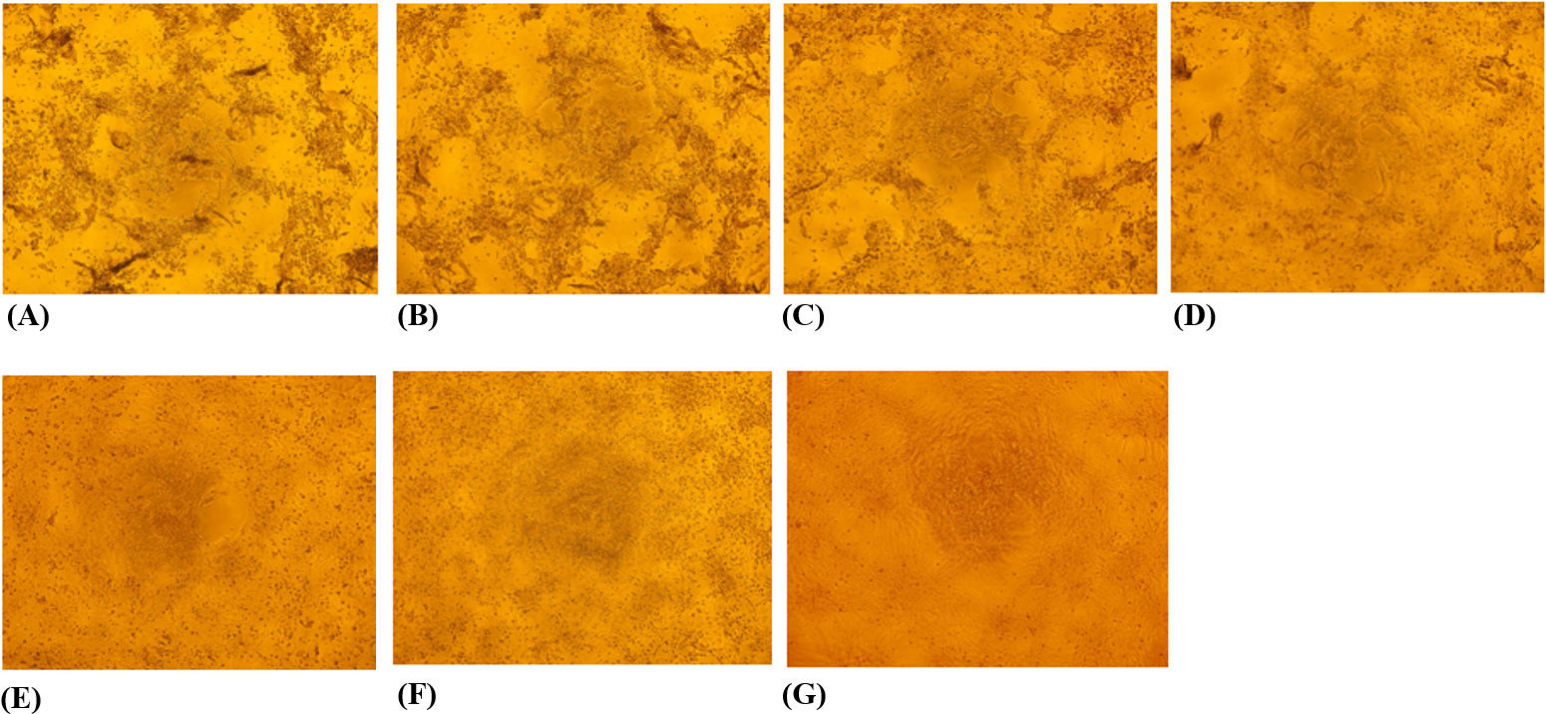
Inhibition of CPE of SFTSV-infected Vero cells by CP-COV03 as seen by 10 × 10 microscopy with CCK-8 agent. The CPE of infected cells was observed microscopically 7 days posttreatment. (A) SFTSV-infected Vero cells without CP-COV03 treatment, (B) SFTSV-infected Vero cells with CP-COV03 0.78 µM, (C) SFTSV-infected Vero cells with CP-COV03 1.56 µM, (D) SFTSV-infected Vero cells with CP-COV03 3.125 µM, (E) SFTSV-infected Vero cells with CP-COV03 6.25 µM, (F) SFTSV-infected Vero cells with CP-COV03 12.5 µM, and (G) Uninfected Vero cell controls.

SFTSV-infected Vero cells were monitored for CPEs on the seventh day posttreatment with CP-COV03. Initially, a dose-dependent reduction in CPEs in CP-COV03-treated SFTSV-infected cells was observed using a 10 × 10 microscope (Fig. 2). The CPE decreased as the drug concentration increased, confirming the dose-dependent antiviral effect of CP-COV03. Subsequently, the cell viability, measured with the CCK-8 assay, increased as the CP-COV03 concentration increased. Significant inhibition of CPE was observed with CP-COV03 at concentrations ≥12.5 µM, with dose-dependent antiviral effects (Fig. 1).

**Fig 3:**
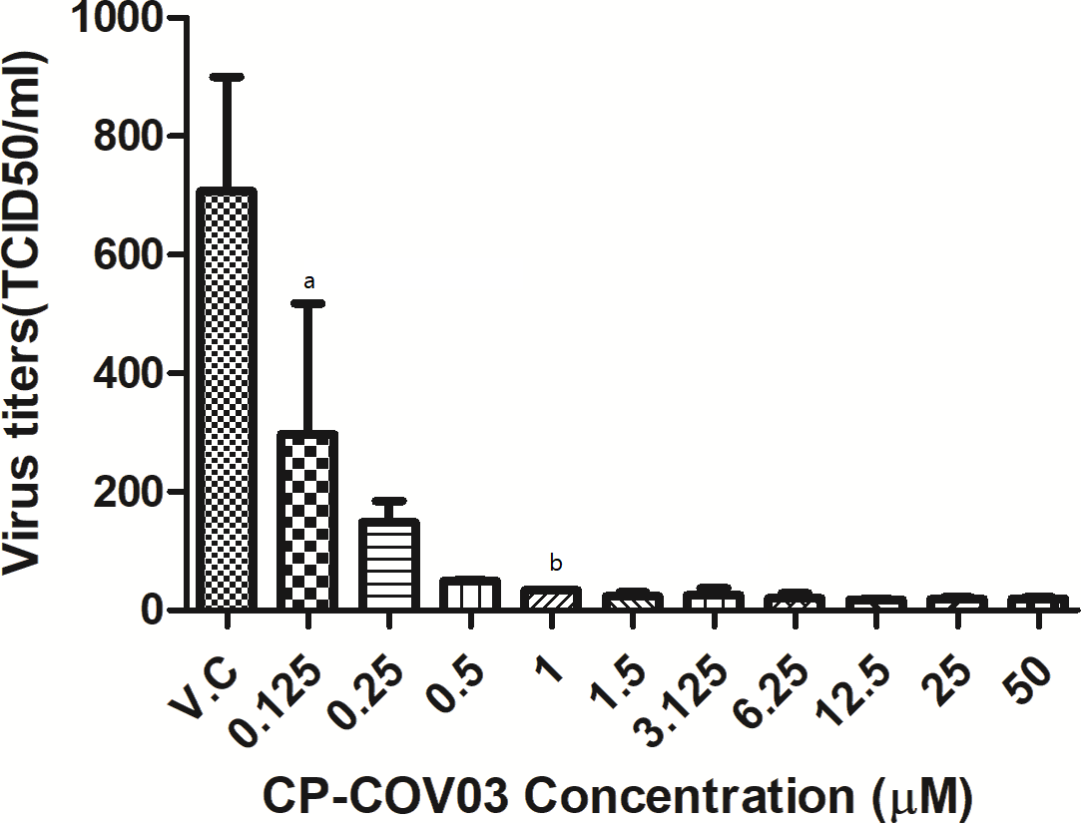
*In vitro* inhibitory effect of CP-COV03 against SFTSV replication in Vero cells as determined by RT-PCR. After their infection with 100 × TCID_50_ of SFTSV for 1 h, Vero cells were treated with increasing concentrations of CP-COV03. Culture supernatants were harvested at the indicated times and their viral RNA titers were assayed using RT-PCR. A one-way analysis of variance with Dunnett’s multiple comparison test (GraphPad Prism 5, GraphPad Software) was used for the data analysis. The data are mean ± standard error of mean. ^a^*p* < 0.01 and ^b^*p* < 0.001 as compared to virus control.

**Fig 4:**
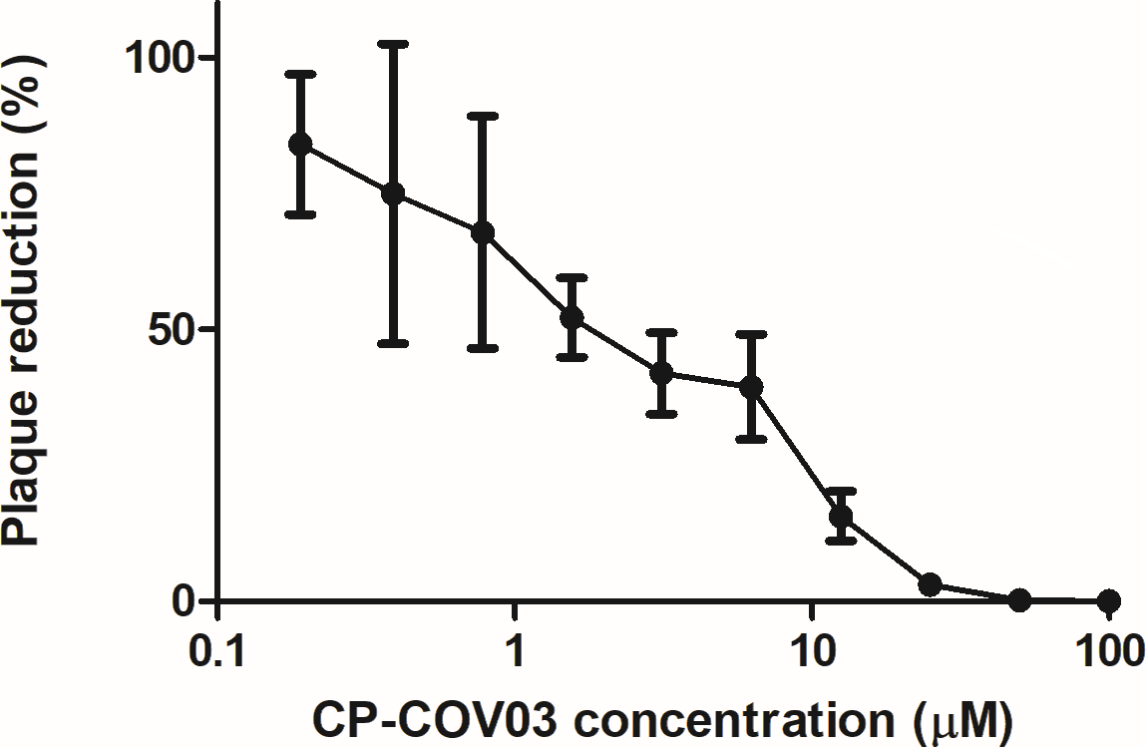
*In vitro* inhibitory effect of CP-COV03 against SFTSV replication in Vero cells as determined by a plaque reduction assay. After their infection with 100 × TCID_50_ of SFTSV for 1 h, Vero cells were treated with increasing concentrations of CP-COV03. The percentage of plaque inhibition relative to the control wells (i.e., without the addition of CP-COV03) was determined for each drug concentration on the second day posttreatment with CP-COV03. IC_50_ was calculated using Sigma Plot (SPSS Inc.) in an Excel add-in ED50V10.

CP-COV03 also showed dose-dependent inhibition on viral RNA replication (Fig. 3). Viral titers were significantly reduced up to a CP-COV03 concentration of 50 µM. In our *in vitro* model of SFTSV infection, the 50% inhibitory concentrations (IC_50_) range of CP-COV03 was below 0.125 µM, as determined from the viral titers of culture supernatants collected on the third day posttreatment of CP-COV03. According to the plaque reduction assay, the percentages of plaque inhibition relative to the control on day two of treatment with CP-COV03 increased in a dose-dependent manner (Fig. 4). The plaque reduction assay showed the IC_50_ of CP-COV03 was 1.893 µM, as determined from the percentage reduction of plaque counts relative to the control wells for each drug concentration on the second day posttreatment with CP-COV03.

## DISCUSSION

We demonstrated that CP-COV03 had a dose-dependent inhibitory effect on the replication of SFTSV in Vero cells. The IC_50_ range was under 0.125 µM according to RT-PCR, which is comparable to the IC_50_ of ribavirin against SFTSV (3.69–8.72 µg/mL [estimated 0.015–0.036 µM]) and lower than the IC_50_ of niclosamide against SARS-CoV (1–3 µM), and the IC_50_ value of niclosamide against SARS-CoV-2 (0.27 µM) (5, 13, 20). Therefore, we suggest that CP-COV03 is a potential therapeutic alternative for SFTSV, given the comparable antiviral efficacy of CP-COV03 against SFTSV to ribavirin in an *in vitro* model and the efficacy of API of CP-COV03.

The 99% inhibitory concentrations of ribavirin are 263, 83, and 78 µM in Vero, Huh7, and U2OS cells, respectively, although they were treated with ribavirin before or during SFTSV infection (21). This inhibitory effect dramatically decreased when Vero cells were treated with ribavirin 3 days after inoculation. SFTSV-infected Vero cells were treated with ribavirin at 24 and 48 h postinoculation and the IC_50_ ranged from 3.69 to 8.72 µg/mL (5). Although there are some differences in the viral strains and treatment procedures, these results suggest that ribavirin is only effective against SFTSV for up to 48 h after inoculation. In this context, the low IC_50_ value of CP-COV03 is an advantage in our study. However, it may also be effective as a postexposure prophylaxis to prevent SFTS or as part of a combination therapy because we treated Vero cells 1 h after inoculation.

Niclosamide, which is the API of CP-COV03, is an Food and Durg Administration (FDA)-approved anthelmintic drug that has been widely used to treat tapeworm infections in humans for decades and is currently on the World Health Organization’s List of Essential Medicines (22). It plays a role in regulating multiple signaling pathways and has moved beyond its role as an anthelmintic. Niclosamide is currently being actively investigated for its efficacy in treating cancer, metabolic syndrome, and bacterial and viral infections (6).

Clinical studies on niclosamide as an anticancer treatment are ongoing, particularly for prostate cancer (23). Niclosamide was administered three times daily (TID) at doses of 500, 1,000, or 1,500 mg in a phase I study of niclosamide plus enzalutamide in patients with castration-resistant prostate cancer (23). The primary objective was to evaluate the safety, and the secondary objective was to assess prostate-specific antigen (PSA) changes and determine the pharmacokinetic profile of niclosamide. The dose of oral niclosamide could not be increased beyond 500 mg TID in this study because the 1,000 mg TID cohort experienced dose-limiting toxicities (prolonged grade 3 nausea, vomiting, diarrhea, and colitis). However, plasma concentrations did not consistently exceed the threshold for growth inhibition in castration-resistant prostate cancer models (24) at the oral dose level of 500 mg TID (24). In contrast, a phase Ib trial of reformulated niclosamide with abiraterone and prednisone in men with castration-resistant prostate cancer shows that niclosamide is tolerable at a dose of 1,200 mg TID, reaching the plasma therapeutic concentration (25). Approved niclosamide formulations have poor oral bioavailability, making it difficult to find an appropriate spot between the maximum tolerable dose and therapeutic concentration. To overcome this, we developed the drug CP-COV03, which significantly improves the bioavailability of niclosamide (17). In a previous study, we reported a proprietary drug delivery technology that improves the bioavailability of niclosamide, enhances the anti-SARS-CoV-2 effect in lung tissues, and improves safety, tolerability, and pharmacokinetic results in healthy adult participants (17).

Since niclosamide is a multifunctional drug, it is challenging to elucidate a single MOA for its antiviral activity. The plausible MOAs of niclosamide’s antiviral activity have been studied across various viruses. One main antiviral mechanism of niclosamide involves the neutralization of endosomal pH and inhibition of viral protein maturation in the Golgi apparatus, particularly in human coronavirus and HCV (26). For MERS-CoV, niclosamide was reported to inhibit replication by enhancing Beclin 1 (BENC1) levels, promoting autophage related gene 14 (ATG14) oligomerization, increasing autolysosome numbers, and affecting autophagic flux in infected cells (14). In the case of flavivirus, one study indicated that niclosamide inhibits Zika virus infection at a post-entry stage, likely during viral RNA replication (27). Another study showed that niclosamide directly inhibits flavivirus NS2B-NS3 interactions, which are essential for flaviviral polyprotein processing (28). For human rhinovirus, niclosamide suppresses viral entry by blocking acidification of the endolysosomal compartments, acting as a proton carrier (29). Regarding the Chikungunya virus, niclosamide not only blocks virus entry via low-pH-dependent fusion inhibition but also prevents cell-to-cell transmission of viral infection (12). For human adenovirus, research indicated that niclosamide inhibits the transport of the virus particle from the endosome to the nuclear envelope (30). Taken together, the prevention of viral entry by altering endosomal pH and the inhibition of viral replication by disrupting autophagy are the plausible MOAs of niclosamide against viral infections (16). Further studies are needed to identify the specific MOA of niclosamide’s antiviral activity against SFTSV.

This study showed IC_50_ of CP-COV03 (<0.125 µM and 1.893 µM, as determined by a real-time RT-qPCR assay and a plaque reduction assay, respectively) for SFTSV are sufficiently low compared to previous reports with other antiviral agents. For example, one study showed that ribavirin reduced SFTSV titers in a dose-dependent manner, with an IC_50_ ranging from 3.69 to 8.72 µg/mL (15.11–35.71 µM), based on viral titers of culture supernatants collected at 24 and 48 h posttreatment (5). Another study reported the 50% effective concentrations of ribavirin, favipiravir, and amodiaquine to be 40.1 ± 16.3, 25.0 ± 9.3, and 19.1 ± 5.1 µM, respectively (31).

Drug repurposing screens during the recent coronavirus disease 2019 (COVID-19) pandemic show that niclosamide has better *in vitro* efficacy than remdesivir against SARS-CoV-2 and is effective in *in vivo* studies (14, 15). A clinical trial showed adding niclosasmide to the standards of care in patients with COVID-19 did not improved survival but reduced time to stay in the hospital compared with controls (32).

In this context, further studies are required to determine whether CP-COV03 with improved oral bioavailability can safely find a therapeutic dose at lower doses for SFTSV. However, we believe that the results of this *in vitro* study will facilitate the *in vivo* and clinical application of CP-COV03 against SFTSV in the future, as niclosamide is effective in controlling symptoms, and is safe in clinical trials of COVID-19. Therefore, confirming the inhibitory effect on viral replication of SFTSV by CP-COV03 in an *in vitro* model is a promising result.

Our study has some limitations. First, the interval between inoculation and treatment was 1 h. Therefore, evaluating the efficacy after a prolonged time interval from inoculation is necessary to evaluate whether CP-COV03 can be used in clinical practice as a treatment beyond post-prophylaxis or combination therapy. This requires further *in vitro* or *in vivo* experiments with adjusted timing of inoculation and treatment. Second, we determined the antiviral efficacy of CP-COV03 by measuring the viral titers in the culture supernatant rather than the amount of cell-associated or intracellular SFTSV. Third, this study did not investigate a specific MOA of niclosamide for SFTSV. The MOA should be addressed by further investigations.

In conclusion, we demonstrated that CP-COV03 exerts antiviral effects by inhibiting viral replication in a dose-dependent manner and could be a promising antiviral agent for SFTSV, given its low estimated IC_50_ value.
